# Supplementary material for: Are carbon emissions trading and green financial instruments synergistic? -Comprehensive quantitative research based on content analysis
Source: PLoS One. 2024 Mar 7;19(3):e0298601. doi: 10.1371/journal.pone.0298601 (PMC10919601; doi:10.1371/journal.pone.0298601)
Supplement: S1 Data — (ZIP) [file pone.0298601.s001.zip › Data/地级市Z_F工作报告.zip!/╜¡╦╒/╤∩╓▌╩╨/└┤╫╘╣1⁄2╓┌║┼í╛┬φ┐╦╩2╛▌═°í┐_2009.docx]

各位代表：

现在，我代表扬州市人民政府，向大会作工作报告，请予审议，并请市政协各位委员和其他列席人员提出意见。

2008年政府工作回顾

2008年，我们在中共扬州市委的领导下，高举中国特色社会主义伟大旗帜，坚持邓小平理论和“三个代表”重要思想，以科学发展观统领经济社会发展全局，依靠和带领全市人民，沉着应对国内外复杂多变的形势，努力克服各种挑战和困难，基本完成市六届人大一次会议确定的目标任务，实现了本届政府工作的良好开局。

一、国民经济保持增长

预计全市实现地区生产总值1580亿元，增长14%左右。人均地区生产总值35400元。财政总收入266.2亿元，其中一般预算收入104.8亿元，分别增长24.6%和22.3%。全社会固定资产投资950亿元，增长32.3%。城市居民人均可支配收入17400元，农民人均纯收入7380元，分别增长15.5%和12%。居民消费价格涨幅4.8%，低于全省平均水平。

工业加快集聚发展。规模以上工业实现产值3518亿元、增加值900亿元、利税270亿元，分别增长35.5%、17%和22.7%。石油化工、汽车船舶、机电装备三大主导产业产值增长38%。“三新”产业产值213.7亿元，增长38.4%。完成工业投资710亿元，增长41.3%。实施亿元以上项目300个，其中10亿元以上项目31个。顺大多晶硅、实友化工、诚德钢管二期等重大项目建成投产。新增产值超10亿元以上企业15家，其中50亿元企业1家。宝胜集团营业收入超过百亿元。新增中国驰名商标12个。全市万元地区生产总值综合能耗下降4%。建筑业总产值1012亿元，增长26%；获鲁班奖两项。

服务业质态进一步优化。服务业增加值561亿元，增长15.1%。扬州港口、石化和公铁水等重点物流园区加快建设。实施《“数字扬州”2010行动计划》，启动无线宽带等8个项目建设。江苏信息服务产业基地（扬州）签约入驻企业20家。信息服务业销售收入55亿元，增长19.6%。全社会消费品零售总额523亿元，增长24.9%。成交额亿元以上市场48家，新增老字号连锁店、加盟店130家。来扬境内外游客1875万人次，旅游收入199亿元，均增长20%以上。房地产业完成投资137.7亿元，增长29.3%。金融机构年末存、贷款余额1551.9亿元和889.4亿元，分别增长23.8%和18.5%。市工艺美术集团获“国家文化产业示范基地”称号。6家服务业集聚区跻身省级行列。

农业生产稳步增长。全面落实各项惠农政策，发放“四项补贴”3.53亿元。粮食总产269.4万吨，连续第五年获得丰收。畜禽、水产及蔬菜稳定增长。新增高效农业面积50.4万亩、设施农业面积5.1万亩，新建规模项目410个；高效农业园区达391个，进园企业（大户）4923个。海峡两岸（扬州）农业合作试验区新引进项目50个。70家市级以上龙头企业实现销售161.6亿元，增长21%。积极推进农产品标准化生产和质量检测，新增“三品”品牌53个。水利、农业开发、粮食流通、供销、农机、气象等工作得到加强。

二、发展活力不断增强

各项改革继续深化。市直工业、商贸流通企业改革深入推进，12家企业改制取得积极进展。市政公用单位改革进一步深化。政府投资管理体制不断完善，部门预算制度改革继续推进。做好企业上市工作，3家企业申报材料已报证监会，1家通过审核；1家企业在境外上市。农村税费、乡镇机构、县乡财政管理体制和义务教育体制改革步伐加快。新增农村“三大合作”经济组织767个。政策性农业保险全面推开。邗江泰和、高邮汇通农村小额贷款有限公司挂牌营业。

科技创新能力明显提升。制订并实施推进科技创新型经济发展的意见。全市获批省级以上科技项目450项、高新技术产品240个。高新技术产业产值865.9亿元，增长49.7%。分别在京沪陕举办“科技创新·产业合作”推介会，签订科技合作项目80个。新增省级以上“一站两中心”13家。首个国家光电产品检测重点实验室落户我市。南京大学—扬州光电、化工研究院和扬州中科半导体照明研发中心加快建设，中科院扬州应用技术研究与转化中心投入使用。启动“百千万人才强市双行动计划”，10名高层次人才获得省专项资助。我市被列为国家知识产权试点城市。

双资利用持续增长。开展多形式招商推介活动，成功举办“烟花三月”国际经贸旅游节。强化跟踪督查和配套服务，促进项目落地、投产达效。新批总投资1000万美元以上项目122个。全年协议利用外资50.7亿美元、注册外资实际到帐17.2亿美元，分别增长48.5%和50.7%。外贸出口45亿美元，增长38%。外经营业额1.85亿美元，增长23%。园区完成基础设施投入69亿元，“八区二园”主营收入占全市工业比重达76%，同比提高4.5个百分点。民营企业注册资本385亿元，增长51.6%。新开工建设5000万元以上项目358个，其中亿元以上项目120个。新增中小企业担保机构5家，注册资本2.39亿元。外事、侨务、台湾事务和区域协作等工作在扩大开放中发挥了积极作用。

三、城乡面貌呈现新变化

重大基础设施加快建设。扬天公路全线建成通车。京杭运河“三改二”一期工程基本完成。南水北调江都截污导流工程、归江河道水利血防工程顺利建成。沪陕高速公路江都至六合段、江海高速公路、安大公路三垛以北段、新淮江公路，以及邵伯和施桥船闸改造、乌塔沟分洪道、第五水厂一期等工程开工建设。苏中江都机场、淮扬镇铁路等重大项目前期工作取得较大突破。

城市建设又有新进展。编制“一体两翼”城市发展空间战略规划和综合交通规划。实施新一轮城市建设和环境提升工程，完成投资116亿元。新改建解放北路、平山堂东路和大水湾步行桥等“七路一桥”，翻建街巷48条。城东客运中心投入运营，万花园二期、文化艺术中心等项目开工建设。基本完成“双东”历史街区“一片十点”修复，启动东关街二期工程。成功举办第二届世界运河名城博览会。积极做好瘦西湖及扬州历史城区申遗工作和大运河联合申遗牵头工作。新城西区、瘦西湖新区、广陵新城开发建设取得新成效。

新农村建设深入推进。完成全市镇村布局规划。市域环路产业带建设积极推进。农村“新五件实事”和“十大工程”投入建设资金5亿元。新改建农村公路506公里、桥梁226座。疏浚县乡河道266条。新增24万人饮用安全水。新建无害化卫生户厕5万座、“一池三改”户用沼气池1.1万个。创建全面小康达标村328个。基本农田和耕地保有量得到有效保护，复垦、整理耕地2万亩，实现占补平衡。机关和企业结对帮扶253个经济薄弱村，农村扶贫工作取得积极进展。

生态环境保护力度加大。深入推进生态市建设。新增污水管网260公里，新建乡镇污水处理厂（设施）13个，城市生活污水处理率达83.6%。70个乡镇工业集中区完成区域环评，162家企业实施清洁生产。化学需氧量、二氧化硫排放量分别下降3.7%和3.1%。绿化造林11.1万亩，森林覆盖率达16%。市区新增绿化面积151万平方米。11个乡镇（街道）通过全国环境优美乡镇考核，创成国家和省级生态村18个。

四、社会保持和谐稳定

民生工作取得新成效。城乡就业同步推进，全市新增就业8万人，年末城镇登记失业率2.9%。就业服务体系渐趋完善。培训就业再就业人员6万人、农村劳动力8.9万人。3万名失业人员实现再就业。新增农村劳动力转移5.7万人。社会保险覆盖面进一步扩大，全市城镇职工基本养老、基本医疗、失业保险覆盖率分别达96%、97%和97%，城镇居民基本医疗保险参保率96%。农民工参加三大保险人数稳步增加。新型农保参保人数35万人。新型农村合作医疗覆盖率97%。被征地农民基本生活保障和“村改居”工作积极推进。稳步增加职工退休金。提高低保户补助标准，城乡低保对象基本做到应保尽保。农村五保户集中供养率达80%。出台特困人群助保办法，完善临时救助机制。加大保障性住房建设力度，对符合条件的低收入家庭提供住房保障。出台公交优先发展的财政扶持政策，新辟、调整线路27条，改造站棚96座，新增公交车200辆；整治8个老小区；建设改造7座农贸市场。市区为民办实事项目基本完成。

各项社会事业持续发展。加强区域教育现代化建设，推进教育均衡发展。全面开展“教育质量效益年”活动，教师素质得到提升。为义务教育阶段学生免除学杂费、免费提供教科书。高中阶段毛入学率稳定在95%以上。整合职业教育资源，办学能力进一步增强。完成佛教文化博物馆等一批文化博览城建设项目。扬州博物馆荣获首批国家一级博物馆称号。新列入国家级非物质文化遗产名录8项。认定并公布104个市级非物质文化遗产和143个市级文保单位。基层文化设施不断完善。开展“百场公益文艺演出”、“周周看扬剧”等活动。扬州评话《王少堂》获第五届中国曲艺“牡丹奖”文学奖。公共卫生服务工作得到加强，城乡社区卫生机构覆盖率分别达91%和85%。苏北人民医院、市第一人民医院和市中医院通过三级甲等医院复审。顺利举办第三届鉴真国际马拉松（半程）赛和市第十一届运动会。计划生育率99.12%。全市有线电视入户率86%，市区基本实现数字电视整体转换。统计、档案、方志等工作扎实开展。

和谐扬州建设迈出新步伐。奥运火炬扬州传递圆满成功，展示了城市新形象和市民新风貌。积极开展文明城市创建活动。未成年人思想道德建设和关心下一代、老龄工作取得新进展。关心发展慈善事业。残疾人救助和保障体系不断完善。平安扬州建设深入开展。全面落实社会治安综合治理各项措施，依法打击各类违法犯罪活动。建立健全社会预警和突发公共事件应急处置机制。认真做好来信来访、人民调解工作。强化安全生产责任制，重视食品药品安全和公共场所监管，有效防范了重特大安全事故的发生。加强国防后备力量建设，积极做好国防教育、人民防空和双拥工作。

五、服务型政府建设不断深入

民主法制建设进一步加强。不断完善社区管理和村民自治制度，重视发挥社会团体、民间组织的作用，保障人民群众依法行使民主权利。制定重大行政事项决策听证办法，推进决策形成、实施等过程的公开。深入开展“五五”普法教育。加大法律服务和法律援助工作力度。坚持依法行政，进一步提升行政执法水平。推行部门权力内控体系建设，规范行政和事业单位办事权。强化行政执法监督和行政复议等工作。加强政府制度化建设，全年制订规范性文件35件。

监督制度不断健全。依法接受市人大及其常委会的法律监督、工作监督，主动接受市政协的民主监督，认真听取各民主党派、工商联、无党派人士和人民团体的意见。坚持与人大

代表、政协委员的联系制度。办理人大代表建议297件、政协提案415件。编制政府信息公开指南和目录，加强信息公开载体建设。市、县政府及市有关部门建立新闻发言人制度。聘请一批政府督查专员。完善对政府重点工作和政务公开的评估监督机制。积极发挥监察、审计等内部监督的作用。高度重视新闻舆论和人民群众监督，政府公开电话和“寄语市长”回复

率均在90%以上。

能力作风建设得到提升。大力开展“能力作风建设年”活动。深入推进行政审批“两集中、两到位”。继续完善便民服务体系。清理审核公共服务项目，取消一批收费项目，规范收费标准816项，服务事项办理时限平均缩短30%以上。试行政府雇员制度。加强公务员培训，提高为民服务本领。认真落实党风廉政建设责任制，坚持勤政为民、廉洁从政。抓好重点领域、重点行业的纠风和反腐败工作，查处了一批违纪违法案件。各位代表，刚刚过去的一年，标志着我国改革开放走过了30年。30年来，我市经济实力显著增强，开放开发突飞猛进，城市面貌日新月异，人民生活蒸蒸日上，扬州发生了翻天覆地的变化。与1978年相比，2008年全市地区生产总值、财政收入、城市居民人均可支配收入和农民人均纯收入分别增加109倍、130倍、54倍和86倍。30年不平凡的历程启示我们：只有始终以解放思想为先导，实事求是、与时俱进，才能克服前进道路上各种艰难险阻；只有始终以发展为第一要务，抢抓机遇、务实奋进，才能不断提升城市的综合实力和竞争力；只有始终以改革开放为动力，砺志创新、永不停步，才能永葆经济社会的勃勃生机；只有始终以改善民生为根本，亲民爱民、惠民富民，才能获得最广泛的群众基础和力量源泉；只有始终以古代文化与现代文明交相辉映为特色，传承文脉、提升品质，才能建设更加富裕、文明、秀美的新扬州。

2008年，我们共同经历了许多大事、难事。我们战胜了特大冰雪灾害，开展了支援抗震救灾和灾区恢复重建工作，完成了奥运火炬传递等任务，特别是下半年我们积极应对经济环境巨大变化带来的影响，保持了经济持续发展、社会和谐稳定。这些成绩的取得，是中共扬州市委正确领导、科学决策的结果，是市人大、市政协有效监督、大力支持的结果，是全市人民齐心协力、攻坚克难的结果。在此，我代表市人民政府，向全市人民，向各位人大代表、政协委员，向所有关心、支持、参与扬州建设和发展的同志们、朋友们，表示崇高的敬意和衷心的感谢！

在肯定成绩的同时，我们也清醒地看到，经济社会发展中还存在一些值得重视的问题。从外部环境来看，全球金融危机的影响还在加深，经济下行的趋势尚未得到有效遏制，这对我市经济增长、企业发展、财政增收、就业再就业、群众收入增加等带来了较大困难。从自身情况来看，优势产业集聚度还需进一步提高，企业增效和创新能力亟待加强，统筹城乡发展的任务还较艰巨，城市功能和品质有待继续提升，资源能源需求和节能减排的压力较大，政府机关在服务意识、工作作风、创新能力和廉政建设等方面还要进一步改进和提升。这些问题都需要我们在今后的工作中，采取更加有效的措施加以解决。

2009年政府工作任务

2009年是全面落实科学发展观的重要一年，是实现“全面达小康，建设新扬州”的关键之年。做好今年工作，既要充分认清经济发展面临的严峻形势，更要切实把握宏观政策调整带来的重要机遇，进一步增强信心，主动应对，克难求进，积极作为。政府工作的总体要求是：认真贯彻党的十七大、十七届三中全会和中央经济工作会议精神，以邓小平理论和“三个代表”重要思想为指导，深入落实科学发展观，进一步扩大有效需求，努力保持经济平稳较快增长；进一步提升科技创新能力，加快转变发展方式；进一步深化改革开放，增强经济社会发展活力；进一步统筹城乡发展，建设更高水平小康社会；进一步改善民生，推动社会和谐；进一步加强政府自身建设，提高公共服务水平，努力保持我市经济社会持续健康发展的良好局面。

全市经济和社会发展的预期目标为：地区生产总值增长12%。财政总收入增长15%，其中一般预算收入增长15%。全社会固定资产投资增长25%。社会消费品零售总额增长16%。城市居民人均可支配收入增长12%左右。农民人均纯收入增长10%左右。居民消费价格涨幅4%左右。城镇登记失业率控制在4%以内。约束性指标为：万元地区生产总值综合能耗下降4%以上，化学需氧量、二氧化硫排放量分别削减3.7%和3.2%。主要抓好六项工作：

一、落实保增长各项措施，推动经济平稳较快增长

积极支持企业健康运行。千方百计帮助企业解决生产经营中的矛盾和问题，提高抗风险、保增长的能力。落实好国家和省出口退税、扶持高新技术企业发展等税费优惠政策，实施增值税转型等改革措施。以财政贴息、以奖代补、信贷担保等多种方式，支持企业结构调整、并购重组和外贸出口。努力保持信贷总量较快增长，贷款增幅力争高于上年和全省平均水平。取消和停征一批行政事业性收费和基金，阶段性调整企业社保缴费标准，降低企业运行成本。强化银企合作、和谐劳动关系、保障要素需求等方面的服务。支持重点企业和利税大户扩大产销、提高效益。引导外贸出口企业加快调整产品和市场结构。促进中小企业担保机构发展，支持做大规模、做强实力，增强对中小企业的服务能力。

切实增加产业项目投入。全年工业投资900亿元，实施亿元以上项目400个，其中10亿元以上项目40个。培植产值千万元以上新增点项目1000个。加快中海造船、顺大多晶硅二期、实友化工二期、东联化学等一批龙头型项目建设步伐。实施100个服务业重大项目，完成投资260亿元以上。拓宽投资领域和渠道，鼓励和引导各类社会资本参与产业项目投资。努力提高项目投资效率，确保一批重点项目投产达效，促进税源经济发展。

大力推进重大基础设施建设。重点实施沪陕高速江都至六合段、江海高速、安大公路宝应段、新淮江公路、扬州港5号码头、京杭大运河“三改二”二期等工程，推进乌塔沟分洪道、淮河入江水道整治等重点水利工程建设。开工建设苏中江都机场、500千伏扬州西输变电、宁启铁路复线及电气化改造等重大项目。积极做好淮扬镇铁路、五峰山过江通道、京沪高速扩容、长江扬镇河段治理等项目前期工作，力争取得重大成效。

进一步扩大城乡消费需求。抓住海峡两岸大三通、长三角区域合作、上海世博会筹办以及国家即将出台“国民休闲计划”等机遇，放大人居城市效应，打造休闲度假特色品牌。办好第19届中国厨师节和第44届全国工艺品、旅游纪念品交易会。吸引更多的境内外游客来扬休闲度假，繁荣旅游消费市场。积极推进核心商圈和专业市场的建设，大力实施放心消费工程。完善县级商业网点规划。进一步培育社区商业和“万村千乡”市场，做好“家电下乡”财政补贴工作，引导和扩大农村消费。落实稳定房地产市场的各项措施，促进房地产业健康发展。

二、调整优化结构，加快转变经济发展方式

大力发展科技创新型经济。完善科技创新体系，着力建设100家重点技术研发机构和10大公共技术服务平台，重点建设“两院两中心”和国家光电产品检测重点实验室，争创国家级太阳能光伏产业基地。推进产学研结合，放大“科技创新·产业合作”活动成效，组织实施一批重大技术攻关和成果转化项目，培育一批科技创新型大企业和科技成长型企业。高新技术产业产值增长30%以上。优化创新人才发展环境，继续实施“百千万人才强市双行动计划”，加快引进、培养高层次技术人才和科技创新领军人才，充分发挥各类人才的作用。大力发展科技服务中介机构。完善科技创新投融资机制。设立市科技创新专项基金，市级财政对科技创新投入不低于1亿元。

培育壮大先进制造业。做大做强石油化工、汽车船舶、机电装备和太阳能光伏等4个千亿级产业，产值增长30%以上。大力推进“三新”产业发展，产值增长30%以上。规模以上工

业增加值增长15%。加快运用信息技术和现代管理技术改造传统产业。支持企业发展具有自主知识产权和关键技术的产品，创省级以上品牌50件，其中国家级8件以上。新批国家级行业标准授权4家。加快市直企业战略重组和退城进园。加强对企业上市的指导，重点跟踪培育上市后备企业20家。推动扬农化工等上市公司再融资。鼓励有条件的企业发行债券。

加快发展现代服务业。推进扬州港口等重点物流园区建设，引进一批国内外知名物流企业。加快“数字扬州”项目建设，建成政府数据资源共享、网络信息交换等中心，推进“无线扬州”二期等一批信息化工程。建设江苏信息服务产业基地（扬州）二期工程，新进园项目20个，争创国家级信息服务外包基地。全年软件与信息服务业实现销售收入65亿元。整合古城、古运河和瘦西湖旅游资源，推进万花园二期、演艺中心等项目建设，打造“双东”、教场、工艺坊等民俗文化街区。全年接待境内外游客2000万人次，旅游收入增长15%以上。大力发展文化创意产业，新创1—2个省级文化产业示范基地。逐步完善融资服务体系，全年新增市级金融机构1—2家，积极引进创业投资基金来扬开展业务。

三、扎实推进农村改革发展，加快构建城乡一体化新格局

深化农村各项改革。全面贯彻十七届三中全会精神，推进农村基本经营、土地管理、农业支持保护、农村金融、城乡一体化发展和民主管理制度改革。规范农村土地承包经营权流转，建立健全流转服务体系和交易平台。进一步推进农村“三大合作”，全年新组建各类合作组织600个，注重提高合作组织运行质态。落实各项涉农补贴，建立健全财政支农资金稳定增长机制。继续深化乡镇机构改革试点。做好县（市、区）农村信用联社改建农村商业银行工作。年内各县（市）至少成立1家农村小额贷款公司。开展村镇银行试点。扩大农业政策性保险覆盖面。

进一步统筹城乡发展。推进城乡规划、产业发展、基础设施、就业保障、公共服务和社会管理一体化。加快沿江沿河开发和市域环路经济带建设，实现“强县强镇、三年倍增”目标，提升县域经济综合实力。实施新农村建设十大工程。新改建农村公路380公里。推进农村电气化建设。实施信息化“一站通”示范村工程。推进中小型水库除险加固和大型灌区改造。新增20万人饮用安全水。新增无害化卫生户厕6万座、“一池三改”户用沼气池1万个以上。加大秸杆还田和综合利用力度。推广清洁能源。加快农村扶贫脱贫步伐。新创建170个全面小康村。江都、仪征创成全面小康达标县（市）。扩大建筑业市场份额，全年施工产值增长15%以上。积极发展农村二三产业，努力增加农民收入。

加快建设现代高效农业。进一步优化种养结构，增强农业综合生产能力。加强115个高效农业重点示范园区建设，打造一批高效农业专业村、特色镇。全年新增高效农业面积40万

亩、设施农业5万亩，其中连片百亩以上的规模园区20万亩。实施6个“万顷良田”试点。大力培植农产品加工龙头企业，提升产业化经营水平。积极推广新品种、新技术、新机具。加大农业综合开发力度。加强农产品质量建设，新增一批无公害、有机农产品。强化动物疫病防控和农作物植保工作。加快建设海峡两岸（扬州）农业合作试验区。

四、扩大开放开发，增强发展活力

加大园区建设力度。推动“八区二园”转型升级，以科技创新型园区、高新技术产业园区为目标，着力在产业链延伸、关键技术研发和产品提升等方面寻求突破，加快建成产学研一体化的先导区、核心区，增强科技带动和辐射功能。各类园区围绕自身特色和优势，加大建设投入，完善科技研发平台、生产生活配套设施等建设，增强承载能力；进一步明晰产业定位，加大引资力度，提高产业集聚发展水平和综合效益。全年园区注册外资实际到帐、重大项目落户数均占全市80%以上。

深入推进对外开放。抓住我国投资环境被外商继续看好的有利时机，坚持量质并举，推进招商选资、招才引智工作，重点吸引产业链高端和关键环节的项目落户扬州，加大技术人才、专业团队和关键设备的引进力度。以企业为主体，突出链式招商、科技招商，加强会展招商和境外办事处招商。办好2009“烟花三月”国际经贸旅游节。全市注册外资实际到帐增长30%以上。优化出口结构，扩大对外工程承包和劳务合作，外贸出口、外经营业额均增长15%以上。

大力发展民营经济。进一步优化发展环境，加大政策扶持力度，引导民营企业加快转型、练好内功、增强活力，培育一批科技创新型企业。进一步放宽市场准入，扩大民间投资的领域，新发展民营企业12000户以上。进一步支持民营企业开展跨地区、跨行业兼并联合，吸引与我市主导产业关联度大的国内民企500强来扬投资。全年民营企业注册资本增长30%，新开工亿元以上项目100个。

五、提升城市品质，打造精致扬州

优化城市规划布局。继续完善市域功能区规划方案。深化“一体两翼”城市发展空间规划，完成城市总体规划修编纲要。编制土地利用整体规划。开展城区地下管网普查，编制完善地下空间利用规划。完成大运河（扬州段）保护规划。加强对重点区域、重点地段的城市设计。完善规划管理制度和执行程序，确保各类规划有效实施。

加强城市建设管理。新建扬仪路、整治扬江路、改造扬冶路，新改建北环路、西三环路等城市主干道，进一步优化城区路网，提高城市通行效率。整治沙施河等4条河道，加快沟通城河水系，美化城市水环境。加快推进新城西区行政商务中心二期和国展中心二期、广陵新城京杭之心、开发区南部新城，以及维扬蜀冈片区、邗江蒋王片区等项目建设。继续做好街景整治和美化、亮化。完善数字化城管配套机制，提高处置效能。深入推进道路保洁、绿化养护等方面市场化运作。着力加强背街小巷和城乡结合部的管理，健全长效机制，提高管理水平。

彰显古城特色。继续加强扬州城大遗址保护工作。深入实施“双东”历史街区保护工程，修建谢馥春、街南书屋等项目。完善汪鲁门古宅、湖北会馆，修缮小盘谷、蔚圃等文保建筑，建设南门遗址展示馆、北门遗址公园，贯通瘦西湖、宋夹城河、长春河、保障湖水上游览线。加强已建项目的保护利用，增添古城生机活力。做好瘦西湖及扬州历史城区申遗工作和大运河联合申遗牵头工作。办好第三届世界运河名城博览会。

改善人居环境。加快“国家生态市”和“国家生态园林城市”创建工作。推进大江风光带等景观绿化带建设，新增造林面积15万亩。市区新增绿化面积100万平方米以上。加快第

五水厂一期、六圩污水处理厂二期工程建设，重视完善地下管网。继续开展大气污染整治、集中式饮用水源地整治等专项行动。仪征、江都创成国家环保模范城。创建20个全国环境优

美乡镇和2个国家级生态村。实施“双百双十”节能计划，鼓励在生产、建设、流通、消费等领域采用节能技术，推进资源节约型、环境友好型社会建设。

六、大力实施民生工程，提高人民群众生活水平

抓好就业再就业和自主创业。实施更加积极的就业促进政策，全年采集就业岗位9万个，实现城镇新增就业4.5万人。完善城乡统一的职业技能培训体系和人力资源市场。重视做好高校毕业生的就业创业工作。加强退役士兵职业技能和农村劳动力培训。新增农村劳动力转移3万人，多渠道为返乡农民工提供就业机会。加大再就业资金筹措力度，扩大失业保险支出范围，加强城乡困难群体的就业援助。发挥创业带动就业倍增效应，建设10个创业孵化基地。

提高社会保障水平。有序开展社会保险扩面工作，企业养老保险净增缴费3万人。推进城乡基本医疗保险全覆盖，进一步完善城镇职工和城镇居民医疗保险体系，提高基本医疗保险待遇。建立健全企事业单位职工和离退休人员收入正常增长机制。提高农民工及部分特殊行业人群参保比例。新型农保覆盖率达60%。提高新型农村合作医疗筹资标准和保障水平。被征地农民即征即保，逐步推动向城镇社会保障并轨。进一步完善住房保障体系，着力提高低收入家庭住房保障水平。积极做好社会救助工作，建立健全城乡低保标准自然增长机制。巩固农村“五保”集中供养水平。对市区70岁以上的“三无”居民发放生活补贴。鼓励发展各类养老服务业。支持发展残疾人社会福利和慈善事业。创建全国无障碍城市。

办好人民满意的教育。加快区域教育现代化进程，促进优质教育均衡发展。着力改善义务教育学校、农村学校办学条件，改善义务教育阶段教师待遇。强化素质教育，提高教育教学质量。加强职业教育骨干学校和重点专业建设。支持扬州大学和地方高校的发展。实施农村留守儿童食宿条件改善和合格幼儿园建设工程。完善教育帮扶体系，办好宏志班。做好普通高中家庭困难学生和中等职业学校学生助学金发放工作。

繁荣文化卫生体育等事业。完善文化基础设施，建设市新图书馆、美术馆、音乐厅。继续推进文化博览城建设，新建扬州学派、马可·波罗纪念馆等一批场馆。做好第三次全国文物普查工作。创作一批群众喜爱的优秀剧目。举办第六届群众文艺新作调演。完善公共卫生服务体系，争创10个省级社区卫生服务中心示范点，城乡社区卫生机构覆盖率分别达100%和

90%以上。新建市级以上卫生镇6个、村30个。建成游泳馆、体校、省中长跑竞走训练基地。办好第四届鉴真国际马拉松（半程）赛。广泛开展全民健身活动。稳定低生育水平，计生优质服务体系覆盖率85%以上。有线电视入户率达90%。深入开展第二次全国经济普查。继续做好民族、宗教、档案、修志等工作。

保持社会和谐稳定。深入推进平安扬州建设，健全社会治安防控体系，积极防范和依法打击各类违法犯罪活动。加强应急指挥中心建设，健全应急管理体系，增强突发公共事件应急处置能力。严格执行安全生产责任制，加强食品药品安全专项整治，强化道路交通、危险化学品、烟花爆竹、特种设备等方面的安全监管，严防重特大事故发生。落实信访工作责任制，完善矛盾纠纷排查化解机制。加强法律援助工作，切实维护群众合法权益。重视防灾减灾，做好支援四川绵竹九龙镇灾后重建工作。加强国防后备力量建设，深入开展国防教育和双拥共建活动。

努力建设人民满意的服务型政府

面对新的形势、新的任务，政府必须全面贯彻科学发展观，坚持以人为本的施政理念，提高把握发展规律、驾驭发展全局的能力，提高转变发展方式、破解发展难题的能力，提高优化服务职能、促进社会和谐的能力。

积极推进管理体制创新。继续推进政企分开、政资分开、政事分开、政府与中介组织分开，改善经济调节，加强社会管理。进一步健全专家咨询、社会公示与听证、合法性审查等制度，完善决策评估机制，有效扩大公众对政府决策的参与度。继续深化行政审批制度改革，拓展服务项目，提升“两集中、两到位”效能。优化财政支出结构，继续加大对“三农”、改善民生、社会事业、科技创新、节能减排等方面的支持力度。完善市区财政体制，继续深化部门预算、国库集中支付等改革。健全政府投资管理体制，加强审计监督，提高财政资金使用效益。全面推进政务公开，拓展公开范围和内容。健全政府信息发布和发言人制度。充分利用政府公开电话、“寄语市长”和部门服务热线，及时答复、解决人民群众的合理诉求。

依法规范权力运行。自觉接受市人大及其常委会法律监督和工作监督，认真落实市人大及其常委会的决议、决定。积极支持市政协履行政治协商、民主监督、参政议政职能。完善与各民主党派、工商联、无党派人士和人民团体的联系制度。认真办理人大代表建议和政协提案。全面落实依法治市的任务要求，深入开展“五五”法制宣传教育活动。健全村民自治和社区居民自治制度，开展100个农村社区建设试点。深入推进行政执法责任制，规范行政处罚自由裁量权和相对集中处罚权。进一步推动部门权力内控体系建设，市直39个部门建成权力内控电子监察系统。强化行政问责和过错追究制。依法开展行政复议、行政应诉和行政执法监督工作，做到有法必依、违法必究，公正执法、文明执法。

进一步提高政府执行力。推进能力作风建设，加强政治理论、法律法规、市场经济和现代科技知识的学习，坚持在学习和实践中拓展科学发展的新思路。认真调查研究，以服务经济、服务企业、服务群众为重点，坚持深入实际、深入基层，不断增强解决实际问题的能力。狠抓各项工作的落实，围绕经济社会发展全局，敢于担当责任，勇于创优争先，强化督查协调，切实提高行政效能。着力解决群众关心的热点、难点问题，办好老小区改造、农贸市场改建、公交优先、公厕改造等方面的实事。

加强政府廉政建设。深入开展反腐倡廉教育，严格执行廉洁自律规定，健全领导干部廉政档案、重大事项申报制度，完善内部制度约束与外部监督相结合的长效管理机制。加强对项目审批权、行政执法权、资金支配权的监督，严肃查处违法违纪行为。建立健全群众评议机制，加强专项治理力度，坚决纠正损害群众利益的不正之风。大力倡导艰苦奋斗、勤俭办事，深化节约型机关创建。努力建设一支素质过硬、作风优良、奋发有为的公务员队伍，树立求实创新、勤政为民、高效廉洁的政府形象。

各位代表，2009年虽然面临较多的挑战和困难，但是，挑战动摇不了我们发展的决心，困难阻挡不了我们前进的步伐。让我们在中共扬州市委的领导下，紧紧依靠全市人民，同心同德，迎难而上，开拓创新，扎实奋进，以新的业绩向建国60周年献礼！
